# Subacute administration of both methcathinone and manganese causes basal ganglia damage in mice resembling that in methcathinone abusers

**DOI:** 10.1007/s00702-019-02110-z

**Published:** 2019-11-30

**Authors:** Andres Asser, Atsuko Hikima, Mari Raki, Kim Bergström, Sarah Rose, Julius Juurmaa, Villem Krispin, Mari Muldmaa, Stella Lilles, Hanna Rätsep, Peter Jenner, Sulev Kõks, Pekka T. Männistö, Pille Taba

**Affiliations:** 1 Department of Neurology and Neurosurgery, University of Tartu, Puusepa 8, 50410 Tartu, Estonia; 2 Faculty of Pharmacy, Center for Drug Research, University of Helsinki, Helsinki, Finland; 3 HUS Medical Imaging Center, Helsinki University Central Hospital, Helsinki, Finland; 4 Faculty of Life Sciences and Medicine, King’s College London, London, UK; 5 Perron Institute for Neurological and Translational Science, University of Western Australia, Perth, WA Australia; 6 Division of Pharmacology and Pharmacotherapy, Faculty of Pharmacy, University of Helsinki, Helsinki, Finland

**Keywords:** Methcathinone, Ephedrone, Manganese, Nigrostriatal damage

## Abstract

An irreversible extrapyramidal syndrome occurs in man after intravenous abuse of “homemade” methcathinone (ephedrone, Mcat) that is contaminated with manganese (Mn) and is accompanied by altered basal ganglia function. Both Mcat and Mn can cause alterations in nigrostriatal function but it remains unknown whether the effects of the ‘homemade’ drug seen in man are due to Mcat or to Mn or to a combination of both. To determine how toxicity occurs, we have investigated the effects of 4-week intraperitoneal administration of Mn (30 mg/kg t.i.d) and Mcat (100 mg/kg t.i.d.) given alone, on the nigrostriatal function in male C57BL6 mice. The effects were compared to those of the ‘homemade’ mixture which contained about 7 mg/kg of Mn and 100 mg/kg of Mcat. Motor function, nigral dopaminergic cell number and markers of pre- and postsynaptic dopaminergic neuronal integrity including SPECT analysis were assessed. All three treatments had similar effects on motor behavior and neuronal markers. All decreased motor activity and induced tyrosine hydroxylase positive cell loss in the substantia nigra. All reduced ^123^I-epidepride binding to D2 receptors in the striatum. Vesicular monoamine transporter 2 (VMAT2) binding was not altered by any drug treatment. However, Mcat treatment alone decreased levels of the dopamine transporter (DAT) and Mn alone reduced GAD immunoreactivity in the striatum. These data suggest that both Mcat and Mn alone could contribute to the neuronal damage caused by the ‘homemade’ mixture but that both produce additional changes that contribute to the extrapyramidal syndrome seen in man.

## Background

Abuse of a ‘homemade’ version of the psychostimulant drug methcathinone (Mcat) causes a persistent and disabling extrapyramidal syndrome (Sikk et al. 2011, 2013; Stepens et al. 2014).This shares some common features with Parkinson’s disease, but has distinct symptoms including early falls, dystonia, severe dysarthria, and a poor response to l-DOPA treatment (Sikk et al. 2011). The precise cause of the neurological symptoms remains unknown. However, the recipe for producing ‘homemade’ Mcat involves the oxidation of pseudoephedrine using potassium permanganate. This produces a solution containing high levels of both Mcat and manganese (Mn) which is then injected intravenously on multiple occasions each day (Sikk et al. 2007). The neurological syndrome that appears in man is accompanied by Mn deposition in the basal ganglia and altered basal ganglia function as assessed by abnormal fluorodeoxyglucose (FDG) uptake (Sikk et al. 2013; Stepens et al. 2014) and decreased striatal D2 receptor binding density (Kessler et al. 2003) shown by MRI and PET investigations.

To date the development of neurological syndrome produced by ‘homemade’ Mcat abuse has been associated with Mn toxicity based on the known effects of exposure to the metal in man (Taba 2013). Mn impairs the release of dopamine without causing a significant loss of nigro-striatal dopaminergic neurons (Guilarte 2013). This would fit with the fact that ‘homemade’ Mcat produced using sodium dichromate as the oxidizing agent, does not appear to have the same toxic effects. However, Mcat itself also causes striatal dopamine release and inhibits its reuptake after both acute and chronic administration (Cozzi et al. 2013) and its potential toxicity on longer term administration is unclear. This raises the possibility that effects of ‘homemade’ Mcat seen in man are either due to Mn toxicity or to the actions of Mcat or to a synergistic interaction. This has never previously been investigated.

In this investigation, we have studied the effects of long-term administration of Mcat or Mn to mice. Importantly, we have compared the effects to those produced by ‘homemade’ Mcat produced according to the recipe used by drug addicts in their kitchens and that contains the same ratio of Mcat to Mn found in the illegal drug mixture. We investigated alterations in motor performance and the effects on dopaminergic neurons in basal ganglia, and changes in postsynaptic dopamine D2 receptor binding SPECT analysis with ^123^I-epidepride.

## Materials and methods

### Animals

Male C57BL6 mice (Harlan Laboratories, The Netherlands) aged 8 weeks were housed in groups of 7–8 in controlled room conditions (temperature, humidity) at 12 h/12 h lighting scheme. In total 60 animals were used for the experiment. They had free access to water and food. The feed was R-70 (LabFor, Sweden) containing 68 mg of Mn per kilogram of feed. Animals were weighed once a week.

### Substances

The following substances were used:

1. Mcat—racemic methcathinone ((RS)-2-(methylamino)-1-phenyl-propan-1-one, Sigma-Aldrich) powder was dissolved in sterile 0.9% saline (B. Braun, Germany).
2. Mn—manganese chloride (MnCl_2_) was dissolved in sterile 0.9% saline.
3. Mcat/Mn—the drug mixture was prepared in a manner closely resembling the methods used by drug abusers and previously described in an article by Sikk et al. (2007): 48 pills containing 60 mg pseudoephedrine hydrochloride (Sudafed, GSK) were stripped of the coating layer and dissolved in 260 ml of boiling tap water. 4 g of potassium permanganate (KMnO_4_) and 4.8 ml of 30% vinegar were added to complete the reaction. The resulting mixture was filtered through filter paper, cooled rapidly to room temperature and stored at + 4 °C to be used as required. A fresh solution was prepared each week.
4. Control—sterile 0.9% saline.

Mcat and Mn content of the drug mixture, as prepared, was measured by a mass spectrometric analysis as published before (Asser et al. 2016). In the drug mixture the average Mcat content was 9.87 mg/ml (range 8.82–10.54 mg/ml) and the average Mn content was 0.688 mg/ml. Animals were dosed intraperitoneally with Mcat, Mn, Mcat/Mn or saline three times a day, 7 days a week for a total duration of 4 weeks. The substances were dissolved with sterile 0.9% saline to deliver a dose of 100 mg/kg of pure methcathinone in the Mcat group, 30 mg/kg of manganese chloride in the Mn group, and 100 mg/kg of methcathinone together with 7 mg/kg of Mn in the Mcat/Mn group. The injection volume was 0.1 ml per 10 g of body weight. Control group animals were intraperitoneally injected with 0.2 ml of 0.9% sterile saline.

### Behavioral testing

Motility box testing was performed pretreatment, and at 2 and 4 weeks. Animals (all groups *n* = 15) were placed singly into plexiglass photoelectric motility boxes measuring 448 × 448 × 450 mm. Covered distance, time in the center area of the box and time in motion were recorded during a period of 30 min immediately after treatment. Group averages were calculated and used for statistical analysis.

### SPECT study

SPECT imaging was carried out after 4 weeks of treatment following the completion of behavioral testing. 48 h after the last drug injection animals (Mcat *n* = 5, Mn *n* = 4, Mcat/Mn *n* = 5, control *n* = 5, selected randomly from respective groups) were anesthetized with isoflurane (4% for induction, 2% for maintenance) in 0.8 l/min medical oxygen as a carrier gas. ^123^I-epidepride (29.9 ± 5.9 MBq; MAP Medical Technologies, Tikkakoski, Finland) in < 200 µl was injected intravenously. Radioactivity in the syringe was measured pre- and post-injection with a dose calibrator (CRC-25R, Capintec Inc., NJ, USA).

SPECT/CT imaging was performed with four-headed small animal scanner NanoSPECT/CT (Bioscan Inc., DC, USA), outfitted with 1.0 mm multi-pinhole mouse apertures. The body temperature was maintained warm throughout the study using a heated animal bed (Minerve, France). Brain SPECT images were acquired 50 min post-injection in 20 projections using time per projection of 270 s resulting in total acquisition time of 23 min. CT imaging was carried out with 45 kVp tube voltage in 180 projections. SPECT images were reconstructed with HiSPECT NG software (Scivis GmbH, Germany) and fused with CT datasets using InVivoScope software (Bioscan).

Reconstructed SPECT images were reoriented and analyzed with InVivoScope software using CT data as a reference. Three-dimensional volume of interest (VOI) was defined around the striatum and cerebellar regions corresponding to D2 receptor-rich and D2 receptor-devoid ^123^I-epidepride brain uptake, respectively. Specific to nonspecific binding ratios (BP) were calculated using the formula: [(counts per voxel in striatum)−(counts per voxel in cerebellum)/(counts per voxel in cerebellum)]. Two independent researchers performed VOI analysis.

### Perfusion and dissection of animals

After the completion of the in vivo ^123^I-epidepride SPECT study and behavioral testing, all experimental animals were euthanized with an intraperitoneal injection of 300 mg/kg thiopental sodium to dissect brain tissues. After the loss of reflexes, a sternotomy was done and the animals were perfused through the left ventricle of the heart with 0.1 M PBS (pH 7.4) followed by 0.1 M PBS containing 4% PFA (pH 7.4). Following dissection, brains were removed and further post-fixed in 4% PFA solution for three days at room temperature and cryoprotected in 0.1 M PBS (pH 7.4) containing 30% sucrose until the tissues sank. Following cryoprotection, the tissues were flash-frozen in liquid nitrogen on dry ice and stored at − 70 °C for further study.

### Immunohistochemical studies

The frozen brain tissue was cut into 20 μm sections using a cryostat (Bright Instrument Co. Ltd, UK) and sections at the level of the striatum and substantia nigra pars compacta (SNpc) were stored in 0.1 M PBS (pH 7.4) containing 0.05% sodium azide at 4 °C as free-floating sections until use.

To determine the dopaminergic neuronal and terminal loss as well as the expression of vesicular monoamine transporter type 2 (VMAT2) and glutamate decarboxylase (GAD 65/67), sections at the level of the SNpc and striatum were stained with different antibodies by peroxidase immunohistochemistry. Three to four sections were stained in each animal. Sections were incubated with 0.3% hydrogen peroxidase in 0.1 M PBS (pH 7.4) to inhibit endogenous peroxidase activity, washed in 0.05% Triton-X-100/PBS and incubated in 20% normal goat serum (NGS)/0.05% Triton-X-100/PBS or 1% bovine serum albumin (BSA)/0.05% Triton-X-100/PBS to block non-specific binding. Sections were further incubated overnight with primary antibodies against tyrosine hydroxylase (TH; rabbit, 1:500; Pel-Freez Biologicals, USA), DAT (rabbit, 1:500; Millipore, UK) or VMAT2 (rabbit, 1:200; Millipore, UK) and glutamate decarboxylase (GAD 65/67; rabbit, 1:500; Millipore, UK). The next day sections were incubated with anti-rabbit biotinylated IgG secondary antibody (diluted 1:200; Vector Laboratories, UK), avidin–biotin complex (Vector Laboratories, UK) and visualized with 0.05% 3,3′-diaminobenzidine and 0.05% hydrogen peroxide. Sections were then mounted onto polyethylene-coated slides (Menzel GmbH & Co KG, Germany), dehydrated in graded ethanol (70%, 98% and 100%), defatted in Histoclear (Fisher Scientific, UK) and cover slipped with DePeX mounting medium (VWR Internationals, UK).

### Cell counting

An Axioskop 40 and AxioCam HRc Rev.3 camera (Both from Carl Zeiss, Germany) were used to take photomicrographs of TH immunostained sections with the AxioVision 4.8 Microscopy Software. The number of TH-immunoreactive cells was determined in the three to four adjacent SNpc sections under magnification 20× in the microscope as described previously by Iravani et al. (2005). Previously we have shown that the counting of dopaminergic neurons at the third cranial nerve provide a reliable anatomical landmark that reflects the extent of cell loss through the entire SNpc (Iravani et al. 2002; Bukhatwa et al. 2009), suggesting that the cell counting at the level of third cranial nerve is an alternative method of determining cell loss in comparison with stereological counting. As each section had 20 μm thickness, all TH immunoreactive cells were counted by changing the magnification of the microscope. Cells that appeared severely deformed were excluded from the counting. Healthy TH-positive dopaminergic neurons had a bipolar shape with dendrites.

The expression level of DAT, GAD 65/67 and VMAT2 in the striatum was determined in the method based on Iravani et al. (2005). Photomicrographs of the whole striatal sections were taken by Canon 500D digital camera in three adjacent sections (+ 0.50–0.62 mm anterior to Bregma) in each animal. Images were analyzed using the Image J software. Relative optical density was assessed for the whole striatum in each animal. Background reading was made from the corpus callosum and anterior commissure. This compensates for slight variations in the intensity of TH immunostaining. Differences in density between the striatum and corpus callosum were taken as the expression levels of each marker.

### Statistical analysis

Means of three sections were calculated for all measured parameters and one-way ANOVA with Newman–Keul’s multiple comparison tests were used for analysis. Two-way ANOVA was used for the analysis of behavioral data. *p* values of < 0.05 were considered statistically significant. Statistical analysis was carried out with GraphPad Prism 6.0 (GraphPad Software, USA).

## Results

Throughout the drug treatment period both Mcat and Mcat/Mn stimulated motor activity, with activity increasing compared to control immediately after dosing. In the motility box, distance covered, time in motion and time spent on rear feet were significantly decreased compared to the control group (Table 1). After 4 weeks of drug treatment a clear suppression in overall motor activity of animals treated with Mcat, Mn, or Mcat/Mn was found.

**Table 1:** Results of motility box testing

	Distance (m)			Time in motion (s)			Time on rear feet (s)		
	Baseline	2 weeks	4 weeks	Baseline	2 weeks	4 weeks	Baseline	2 weeks	4 weeks
Control (*N* = 15)	103.2 (94.9–111.5)	84.4 (77.2–91.7)	85.3 (77.3–93.4)	623.4 (582.8–664.0)	549.8 (512.7–586.8)	553.4 (510.2–596.5)	150.9 (129.0–172.8)	144.2 (111.1–177.3)	114.9 (94.8–135.0)
Mcat (*N* = 15)	104.9 (97.6–112.2)	101.6 (79.7–123.5)	38.8 (22.2–55.4)***	624.9 (584.9–665.0)	600.2 (481.9–718.5)	260.4 (160.8–360.0)***	159.3 (147.1–171.6)	121.8 (88.55–155.1)	52.8 (19.5–86.11)**
Mn (*N* = 15)	92.0 (81.5–102.4)	68.4 (57.1–79.7)*	63.5 (52.7–74.3)**	564.9 (505.9–623.8)	448.5 (379.5–517.6)**	419.5 349.8–489.1)**	126.3 (108.2–144.3)	74.5 (53.8–95.1)***	62.6 (43.6–81.7)***
Mcat/Mn (*N* = 15)	108.4 (96.9–119.9)	110.3 (84.6–135.9)**	62.3 (52.0–72.6)***	648.5 (588.6–708.5)	655.6 (529.8–781.5)*	413.7 (351.3–476.2) ***	158.3 (130.8–185.9)	123.3 (99.4–147.1)	74.9 (47.5–102.2)

Motion was measured for a 30 min period immediately after the injection. All values are group means with 95% CI as compared to the control group at the same time point

*p < 0.05; **p < 0.01; ***p < 0.001 compared to control

Weight of the animals did not change significantly during the 4 weeks. The mean weight by treatment groups were control 22.8 g (95% CI 21.9–23.7), Mcat/Mn 23.3 g (95% CI 22.4–24.2), Mcat 22.7 g (95% CI 21.5–23.9) and Mn 22.1 g (95% CI 21.2–23.1) at baseline and control 25.2 g (95% CI 24.5–25.9), Mcat/Mn 25.2 g (95% CI 23.8–26.7), Mcat 24.3 g (95% CI 23.1–25.5) and Mn 22.2 g (95% CI 21.4–23.1), respectively, by the end of the 4 week treatment period.

**Fig. 1:**
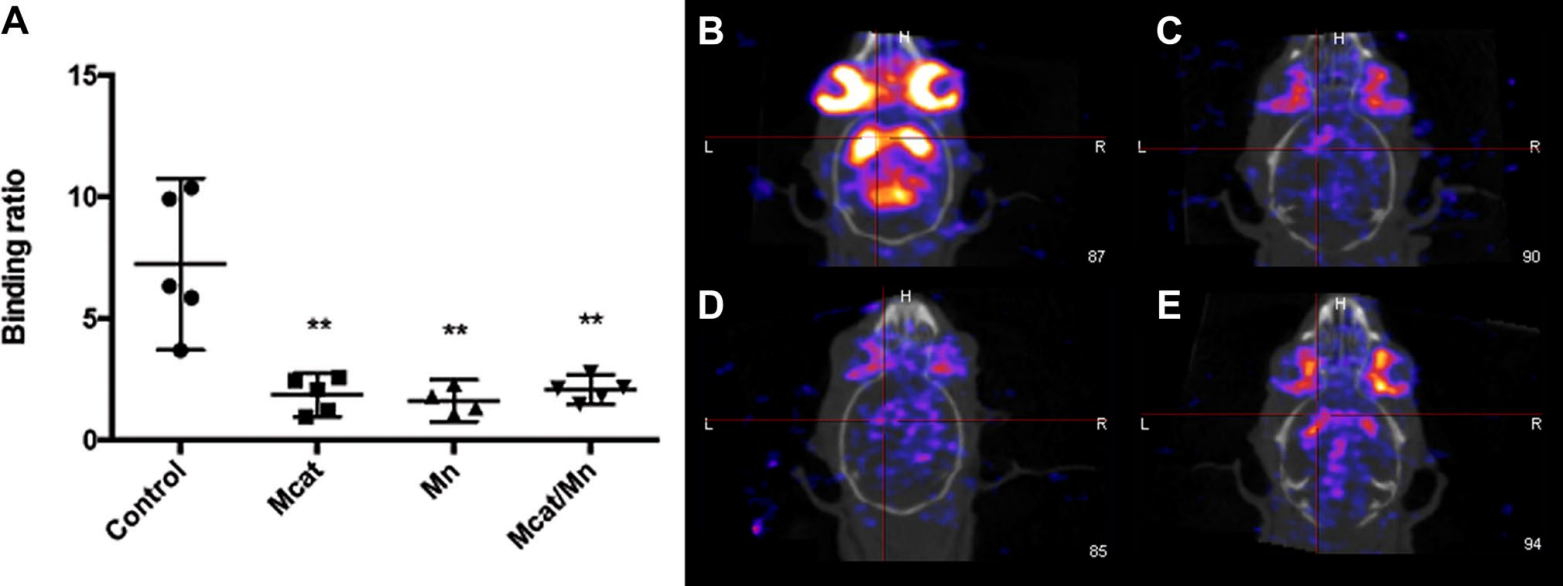
Binding ratio of ^123^I-epidepride uptake and SPECT images of mouse striatum. SPECT imaging demonstrates a significant decrease in epidepride binding in all treatment groups. **a** Mean binding ratios with 95% CI values of controls (*N* = 5), Mcat (*N* = 5), Mn (*N* = 4); Mcat/Mn (*N* = 5). **p < 0.01 compared to control (Newman–Keul’s test). A single illustrative SPECT image from each group **b** control; **c** Mcat; **d** Mn; **e** Mcat/Mn. The red crosshair is placed on the striatum

SPECT imaging demonstrated a significant decrease in specific to nonspecific epidepride binding ratio in Mcat (1.85; 95% CI 0.9–2.7), Mn (1.61; 95% CI 0.7–2.5) and Mcat/Mn (2.06; 95% CI 1.5–2.7) groups compared to the controls (mean 7.22; 95% CI 3.7–10.8; details shown in Fig. 1, left). Representative SPECT images of the anatomical regions are shown in Fig. 1 (right panels).

**Fig. 2:**
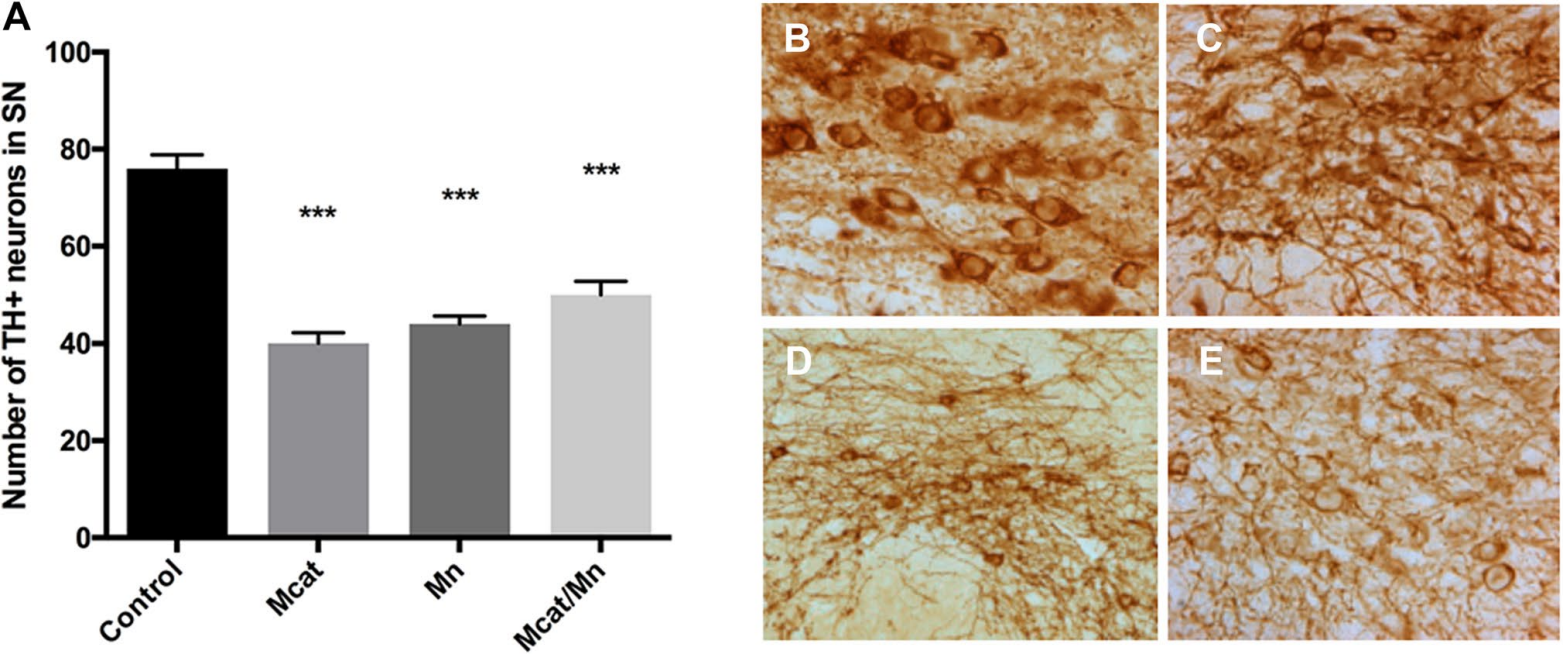
Effect of Mcat, Mn and Mcat/Mn administration on the number of TH-positive dopaminergic neurons in the SNpc. Immunohistochemical staining shows a loss of dopaminergic cells in the SNpc as demonstrated by a decrease in the number of TH positive stained neurons per section. **a** Data are presented as mean with 95% CI. For controls *N* = 7, Mcat (*N* = 12), Mn (*N* = 10) and Mcat/Mn (*N* = 12). ***p < 0.001 compared to the naïve animals (Newman–Keul’s). **b**–**e** An illustrative image of the TH stained cells from a single animal in each group is shown on the right. **b** Control; **c** Mcat; **d** Mn; **e** Mcat/Mn

**Fig. 3:**
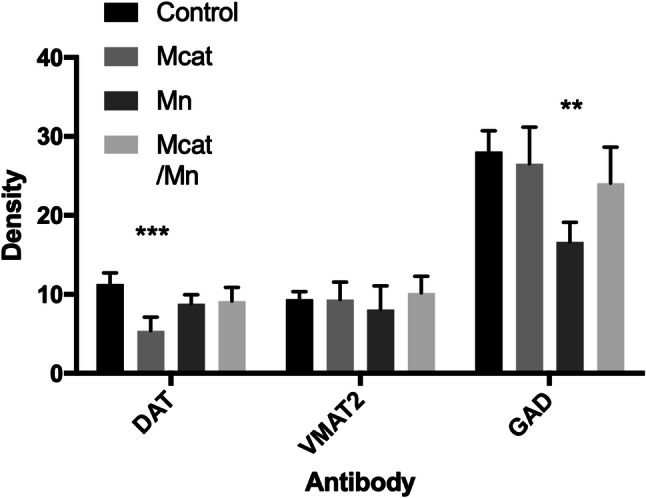
Expression levels of DAT, VMAT2 and GAD 65/67 in the striatum in Mcat-, Mn- and Mcat/Mn-treated animals. Immunostaining of the striatum was measured as optical density using Image J. ***p < 0.001 compared to control animals (Newman–Keul’s). Control (*N* = 10), Mcat (*N* = 6), Mn (*N* = 4) and Mcat/Mn (*N* = 7)

Results of the immunohistochemical staining studies are summarized on Figs. 2 and 3. TH positive cell counting showed a significant decrease of overall cell density in the SN in Mcat, Mn and Mcat/Mn treated animals (Fig. 2). There was a decrease in DAT expression in Mcat-treated animals as compared to the control group (Fig. 3). GAD65/67 staining was reduced by the administration of Mn alone. There was no change in VMAT2 expression following any of the treatments.

## Discussion

We analyzed the effects of two potentially toxic compounds, Mcat and Mn, in a homemade drug based on pseudoephedrine conversion. While Mcat is the desired oxidation product, Mn is generated in a free form from a major ingredient, potassium permanganate. The onset of a parkinsonian syndrome in individuals abusing this cocktail has previously been attributed to Mn toxicity since occupational exposure to the metal is associated with the occurrence of similar motor symptoms (Lucchini et al. 2009; Sikk et al. 2007). In addition, a similarly acting Mcat mixture prepared using sodium dichromate, instead of potassium permanganate does not lead to evident neurological sequelae based on short term exposure (Emerson and Cisek 1993). However, neither the conclusion that Mn alone is responsible for the syndrome occurring in man nor the potential role played by Mcat has been tested. We now show in mice that both components of this drug cocktail, Mn and Mcat, contribute to its toxicity.

The abovementioned syndrome in itself, although devastating for the affected population, is less known throughout the world. At the same time, the use of methcathinone is part of a larger epidemiological trend of synthetic cathinone abuse. The mixture of manganese and methcathinone, also termed ephedrone in literature, is mainly reported as case series or case reports and larger studies are lacking (Sikk and Taba 2015).

Administration of Mcat, Mn and a “home-made” mixture containing the two led to similar and consistent changes in motor function. As expected, there was an immediate acute response to each injection of Mcat alone or in combination with Mn over the 4-week period, in the form of stereotypical movements lasting 30–60 min. This effect has been described in relation to Mcat as well as other psychostimulants (Anneken et al. 2017). But importantly, both components (Mcat and Mn), alone or in combination, equally suppressed overall motor activity by the end of the 4-week treatment period as shown in the motility box. An interesting observation was that this effect was, if anything, more evident with Mcat alone than with Mn alone or Mn in combination with Mcat. Overall, the changes observed in motor function reflect the development of the extrapyramidal syndrome as seen in man. The described locomotor changes are in concordance with previously published literature (see the excellent review by Angoa-Perez et al. (2017).

The alterations in motor function and a marked decrease in the number of TH immunoreactive cells in substantia nigra is in good correlation. The decrease in cell number occurred after the administration of Mn or Mcat or a combination of the two. A similar change following Mn administration was described previously in mice (Stanwood et al. 2009) but this is the first time that this has been shown following Mcat treatment. The decrease presumably reflects a loss of dopaminergic cell bodies although a reduction in TH expression cannot be excluded. Psychostimulants are known to be directly toxic to nigral dopaminergic cells. For example, Ares-Santos et al. (2014) demonstrated a marked reduction in the number of nigral dopaminergic neurons together with changes in locomotor activity in response to methamphetamine administration. The overall conclusion from the examination of nigral tissue, would be that both Mn and Mcat exert toxicity and that this effect is reflected in the combination of these drugs and explains the persistence of the motor deficits seen in man.

More puzzling are the changes in the markers of presynaptic function of dopaminergic neurons in the striatum. It might have been expected that there would be reductions in VMAT2 and DAT that reflected the loss of nigral dopaminergic cells, but this was not the case. No changes in VMAT2 occurred in any treatment group and only Mcat given alone decreased DAT expression. These findings suggest that presynaptic dopaminergic function was occurring relatively normally despite substantial nigral cell loss. The explanation may be that, at the timepoints studied, there was on-going nigral TH positive cell degeneration that had yet to be reflected in changes in striatal markers. Alternatively, it may be that remaining dopaminergic terminals in the striatum have upregulated levels of VMAT2 and DAT to compensate for the losses that had already occurred. Why DAT should only be decreased by Mcat treatment while there is no effect of a combination of Mcat and Mn is a subject for further investigation. If we hypothesize that the toxicity of Mn is preferentially postsynaptic (e.g. GABA neuron) and that of Mcat presynaptic (dopamine neurons), the dose differences may also become meaningful. Doses in the home-made mixture may simply be too low to cause measurable changes. Mn alone has been shown to alter DAT functioning. A study by Roth et al. (2013) for example suggests that Mn induces DAT internalization and thus leads to disruption of dopamine signaling. However, this situation reflects the results of SPECT imaging studies in patients abusing the Mcat and Mn cocktail where presynaptic markers were unaltered despite the presence of marked neurological symptoms (Sikk et al. 2010).

Post-synaptic changes in I^123^epidepride binding to post-synaptic D2 receptors in the striatum on SPECT imaging were marked in animals treated with Mcat alone, Mn alone or a combination of the two. We have assumed that the changes in I^123^-epidepride binding represent a decrease in receptor number and not competition with Mcat for receptor occupancy. Mcat should be cleared from the body by the time of SPECT scanning based on its plasma half-life. The psychomotor effects of another closely related derivative of Mcat, mephedrone, can be measured for 2 h after administration but its plasma concentrations become undetectable 5 h after intravenous administration (Martinez-Clemente et al. 2013). In addition, ^123^I-epidepride has such a high affinity for D2 receptors (Km about 50 pM) that Mcat, having a much lower affinity (micromolar), would be unlikely to displace it (Tibbo et al. 1997; Kornhuber et al. 1995). Presuming this to be the case, there are two potential explanations for these findings. The first would be that there is downregulation of post-synaptic D2 receptors reflected in decreased numbers of epidepride binding sites as a result of receptor internalization. Such a decrease would lead to decreased motor function as previously observed in mice after D2 receptor block or absence (Klinker et al. 2013). The alternative explanation is that there is a loss of striatal cells bearing D2 receptors as a result of the toxicity of Mcat and Mn. This would be consistent with previous studies that have specifically investigated the toxic actions of Mn (Stanwood et al. 2009). D2 receptor bearing interneurons are mostly GABAergic but also cholinergic and glutamatergic neurons are important. We found decreased GAD, a marker of GABA neurons, only in the Mn group. The striatal cells that have D2 receptors on their cell bodies are largely making up the indirect output pathway to the external segment of the globus pallidus. Cell loss would disrupt the striato-thalamo-cortical loop controlling voluntary movement and explain the changes seen in motor function in both man and animals. A strong argument for the importance of postsynaptic damage can be derived from the clinical poor l-DOPA response of the patients. If there is a substantial damage of the cells bearing D2 receptors, the target of l-DOPA therapy is reduced.

One of the main limitations of the present study includes the timing of experiments (locomotor effects, immunohistochemical and SPECT studies). It could be argued that a longer experiment duration could have resulted in more significant changes. While this is true, it was our goal to demonstrate the presence of abovementioned alterations rather than quantify them in detail. In addition, originating from the clinical practice, there are reports of patients suffering from markedly severe symptoms after only a short period of using the drug.

## Conclusions

Based on our results, we conclude that both components of the Mcat and Mn cocktail can exert toxic actions that disrupt dopaminergic function and striatal output that leads to the generation of an extrapyramidal syndrome. While both lead to altered function of the basal ganglia, it is in all probability the addictive properties of Mcat that drive the frequent daily abuse of the cocktail that then inflicts cellular damage. These findings are of importance as despite the best efforts, there has been no detailed examination of post-mortem brain material from the addict population that would have allowed the uncovering of pathophysiological mechanisms responsible for their neurological symptoms to be uncovered.
